# Occlusal records in the production of mounted study casts for tooth wear planning and management

**DOI:** 10.1038/s41415-023-5674-3

**Published:** 2023-03-24

**Authors:** Callum Cowan

**Affiliations:** Consultant in Restorative Dentistry, Liverpool Dental Hospital, Pembroke Place, Liverpool, L3 5PS, UK

## Abstract

This article forms part of a series looking at management of patients with tooth wear. Articulated study casts can be essential in assisting the clinician to plan and communicate proposed treatment to the dental technician and patient. Their production is often seen as straightforward, but a lack of attention to detail can quickly lead to articulated casts that do not replicate the patient clinical presentation. This in turn will lead to inaccurate planning and potentially a suboptimal treatment outcome. This article discusses a collection of the clinical records needed to produce accurate articulated study casts, which can be utilised for tooth wear planning. It also aims to present the evidence base for the recommendations outlined.

## Key points

- Articulated study casts can form an integral part of the treatment planning process for patients with complex tooth wear.
- The stages to produce accurately mounted study casts include recording impressions to achieve high-quality study casts, recording a facebow, recording a centric relation record and lab mounting. Each stage needs to be carried out with the greatest of care to ensure consistent results.
- After the records have been collected and processed by the laboratory, it is essential that the mounted study casts are verified for accuracy. Accuracy is by no means a guarantee and errors can occur at any point. This can result in mounted study casts that do not replicate the tooth contacts found clinically.

## Introduction

Articulated study casts can form an integral part of the treatment planning process for patients with complex tooth wear. The use of analogue techniques to produce articulated study casts is well documented.^1^^,^^2^^,^^3^^,^^4^^,^^5^^,^^6^^,^^7^ The use of digital techniques will be discussed in a separate article within this series.

Articulated study casts serve as a medico-legal record of the patients' pre-treatment presentation. They can be used to examine the static and dynamic occlusal contacts without muscular guarding or the visual limitation of the soft tissues. Duplicated study casts can be modified with mock equilibrations, tooth removal, or additive waxing, to simulate proposed treatment plans. They are also useful in the production of guides and stents. The production of articulated study casts comes at significant expense and must provide added value. They are not always necessary but are invaluable when executing complex tooth wear cases.

The stages to produce accurately mounted study casts include:

- Recording impressions to achieve high-quality study casts
- Recording a facebow
- Recording a centric relation record (CRR)
- Lab mounting and verification.

This article will aim to outline a simple and evidence-based summary of how to execute the stages above to a high standard.

## Stage 1: recording impressions to achieve high-quality study casts

Alginate impression material in a universal stock tray will provide sufficient accuracy to produce high-quality study casts. Alginate provides a cheap and well-tolerated impression material. However, an understanding of the material's limitations is essential if it is to be used consistently.

### Storage

**Fig. 1:**
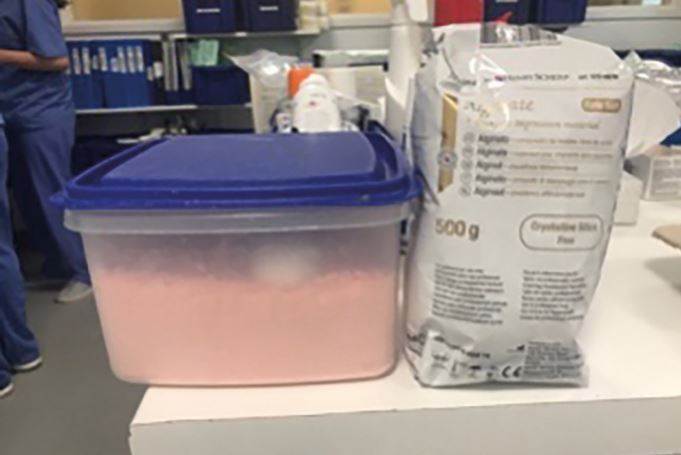
Alginate stored in bulk in an airtight container

Alginate is most commonly bought in bulk. After opening, the loose alginate is stored in an airtight container (Fig. 1). It is essential that alginate stored like this is shaken thoroughly before use. This serves two purposes. Firstly, it distributes the top layer and moisture-compromised alginate evenly throughout the mix. Secondly, it distributes the alginate evenly by weight. Alginate can be measured by weight or volume. Depending on how densely packed the alginate is, a single scoop by volume can vary considerably in weight. By shaking the alginate tub before measuring, the mix will be more homogenous.

### Handling

By altering the powder-to-water ratio when mixing alginate, it is possible to manipulate the consistency. For the best results, it is best to maintain the volume of powder and vary the amount of water. Altering the powder-water ratio by up to 25% does not affect the accuracy of the impression.^8^ Setting time of alginates can vary considerably between manufacturers. Most setting times are based on 22 °C tap water and average 2-3 minutes. By increasing or decreasing the temperature of the water, the setting time can be manipulated. There is some evidence to suggest that higher temperature water results in less dimensionally accurate impressions. For the best results, cold water is recommended.^9^^,^^10^

### Tray selection

The tray is the foundation of an accurate impression. Classically, metal rim lock trays were recognised to be the gold standard. This was born out of concerns about the rigidity of newer plastic stock trays, which were thought to flex and then rebound on impression removal. In reality, the studies that have investigated this have been concerned with high viscosity silicone putty, not alginate.^11^ When using alginate, universal plastic stock trays provide adequate rigidity.

**Fig. 2:**
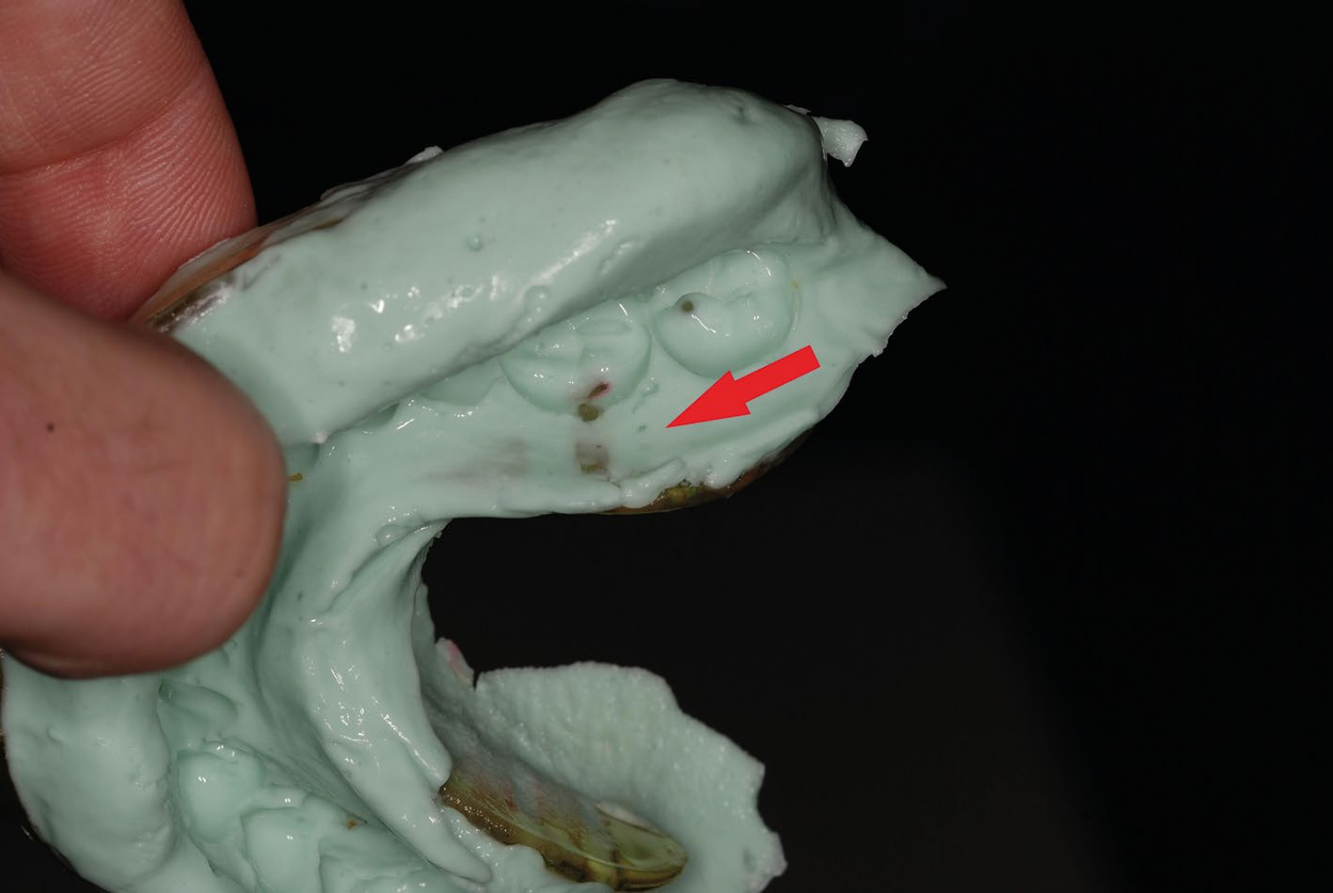
Red arrow points to perforations through alginate, which can lead to inaccurate impressions

**Fig. 3:**
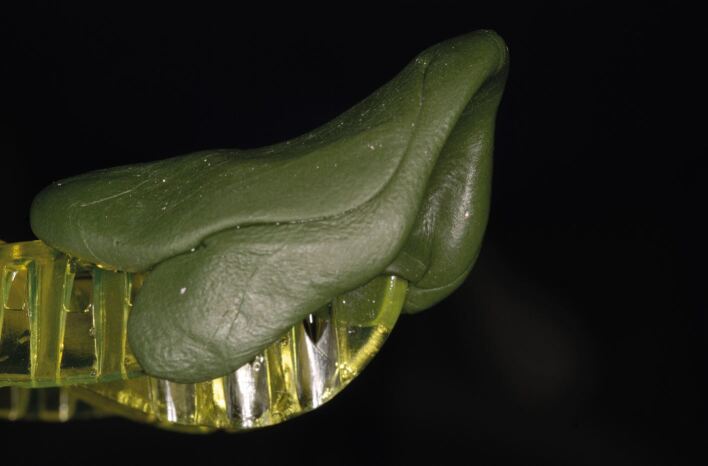
A lower stock tray with inadequate extension which has been modified with greenstick to provide a rigid surface for the impression material

Sizing the tray appropriately is essential. There should be good material support around the full arch and 3-5 mm clearance from the teeth around all aspects of the tray to allow adequate alginate thickness. Tray perforations through the alginate can lead to inaccurate impressions and ideally should be avoided, particularly in key areas (Fig. 2). Stock trays of adequate width will often not be long enough to capture the full length of the arch. In an attempt to capture the most posterior teeth, the clinician will often seat the tray too far back, resulting in the anterior teeth perforating through the impression material as the tray is rotated upwards. A better technique is to modify the stock tray to increase its length with a rigid material, such as an impression compound (Fig. 3). It can be tempting to use wax for this purpose, as it is cheap and fast to use, but it is flexible and can be easily distorted.

**Fig. 4:**
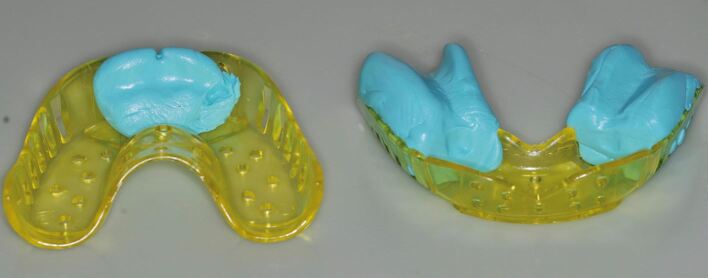
Alginate is best supported to prevent sagging. In this example, putty has been used

Special attention needs to be paid to large edentulous saddles or high-vaulted palates. Alginates do not self-support well and can sag before the gelation phase is complete. The tray is best augmented with compound or putty in these areas to prevent this (Fig. 4).^12^

**Fig. 5:**
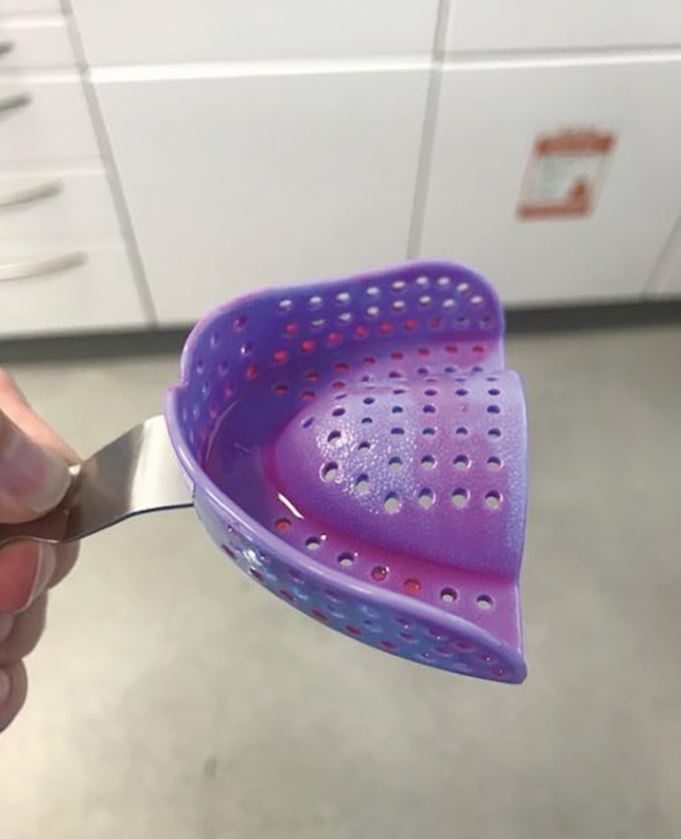
Pooled adhesive acts like a lubricant and must be air thinned or applied in a thinner section

Mechanical retention is provided in stock trays by perforations. This may not be sufficient to ensure impression accuracy. The use of alginate adhesive is recommended. *In vitro* studies have demonstrated that it can improve the dimensional accuracy of alginate impressions.^13^^,^^14^ It is essential that pooled adhesive is not left in the tray as this can act as a lubricant (Fig. 5). Attention to detail and ensuring that the adhesive is wrapped 2-3 mm on to the exterior of the tray will also help.

### Mouth preparation

**Fig. 6:**
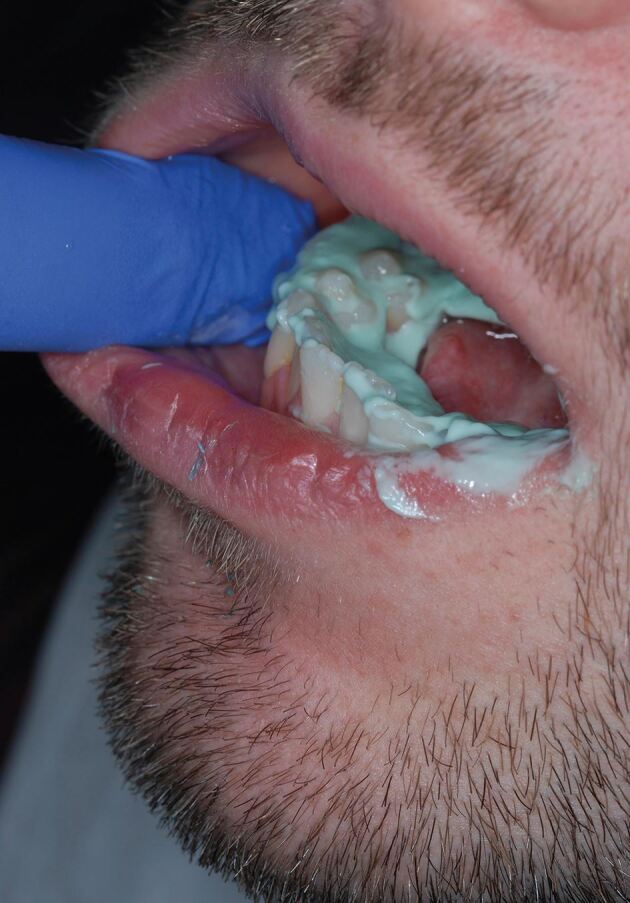
By smearing alginate into fissures before the main impression is taken, it is possible to capture more surface detail

**Fig. 7:**
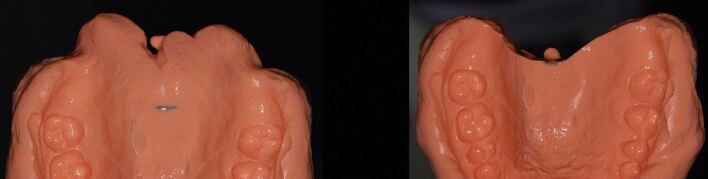
Excess alginate has been trimmed from the posterior border of the tray with a scalpel to reduce the chance of deformation during transport

Mouth preparation should be carried out before making your alginate impression to achieve enhanced surface detail.^15^ The teeth should be gently dried with suction or gauze. Over-drying with 3-in-1 air should be avoided. If the teeth are over-dried, the alginate can form a chemical bond to the hydroxyapatite crystals. This happens most commonly if the pellicle is removed from the tooth when multiple sequential impressions are taken. It can be beneficial if alginate is smeared deeply into the occlusal fissures of the teeth (Fig. 6). This can reduce blebs and air blows. After a full set, the alginate impression should be removed with a sharp snap movement. Gentle wiggling or gradual unseating can cause viscous flow and limit the elastic recovery and accuracy of the impression. The removal of any unsupported excess alginate with a scalpel will reduce the chance of distortion, as the impression is packed for transport. This trimming of the alginate also allows an opportunity to check that the alginate is properly adhered to the tray (Fig. 7).

### Disinfection and preparation for transport

A standard alginate impression is 85% water and is subject to ready release (syneresis) and absorption (imbibition) of water. Special attention has to be paid to impression disinfection, storage, transportation and the time delay until pouring, to prevent suboptimal performance.

The disinfection of an alginate impression can be completed by either immersion in a disinfectant bath or soaking with a disinfectant spray. Both have been shown as effective and achieve cross-infection control. The effect of disinfection on dimensional accuracy has been extensively researched. A recent critical review of the literature demonstrates that there are negligible effects on dimensional accuracy of the impression as long as the manufacturer's instructions are followed.^16^^,^^17^

It is commonly quoted that distortion of an alginate impression can start to develop in as little as 12 minutes after removal.^18^ This has led to the belief that an alginate impression needs to be cast as soon as possible after capture. This has been disputed in other publications and largely depends on the alginate being used.^19^^,^^20^ There have been huge advances in alginate in recent years, with some manufacturers claiming their alginate can maintain dimensional stability for as many as seven days. The evidence would support the dimensional stability of these newer extended pour alginates.^21^ That said, we cannot expect standard alginates to perform in this way. It is essential that these are poured as soon as possible.^22^ If there is likely to be periods of over 24 hours between recording and pouring of your impression, an extended pour alginate or addition cure silicone should be considered.^23^

## Stage 2: recording a facebow transfer

**Fig. 8:**
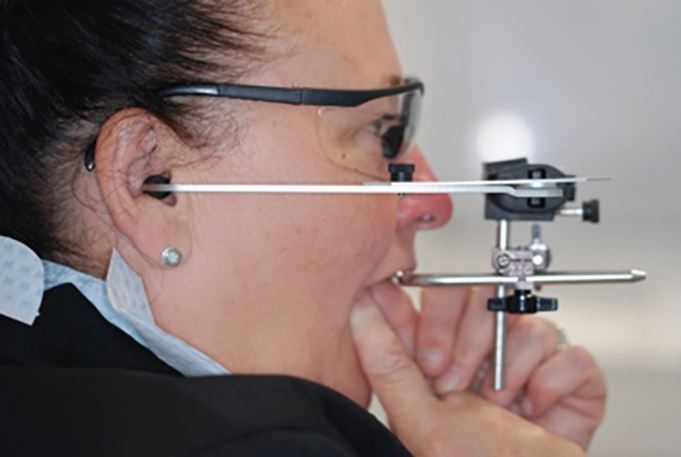
A patient having a Denar Slidematic Facebow completed

The importance of the facebow transfer can be conceptionally difficult to grasp. A facebow allows us to record the spatial orientation of the maxilla to the terminal hinge axis clinically and then transfer this information via the mounting process to an articulator (Fig. 8).

**Fig. 9:**
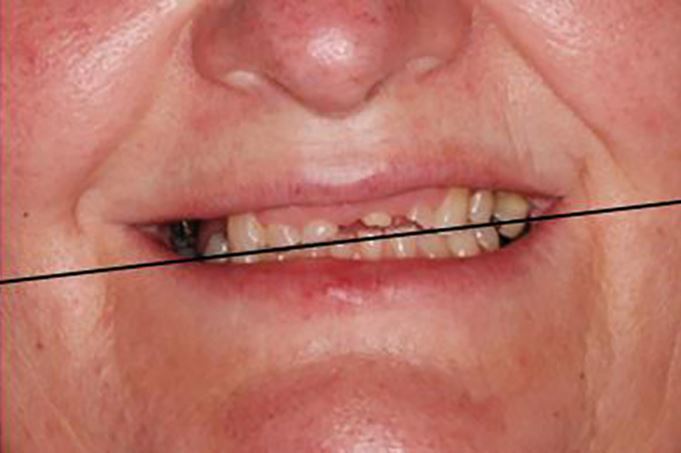
Facebows can be used to communicate cants in the incisal and occlusal plane

**Fig. 10:**
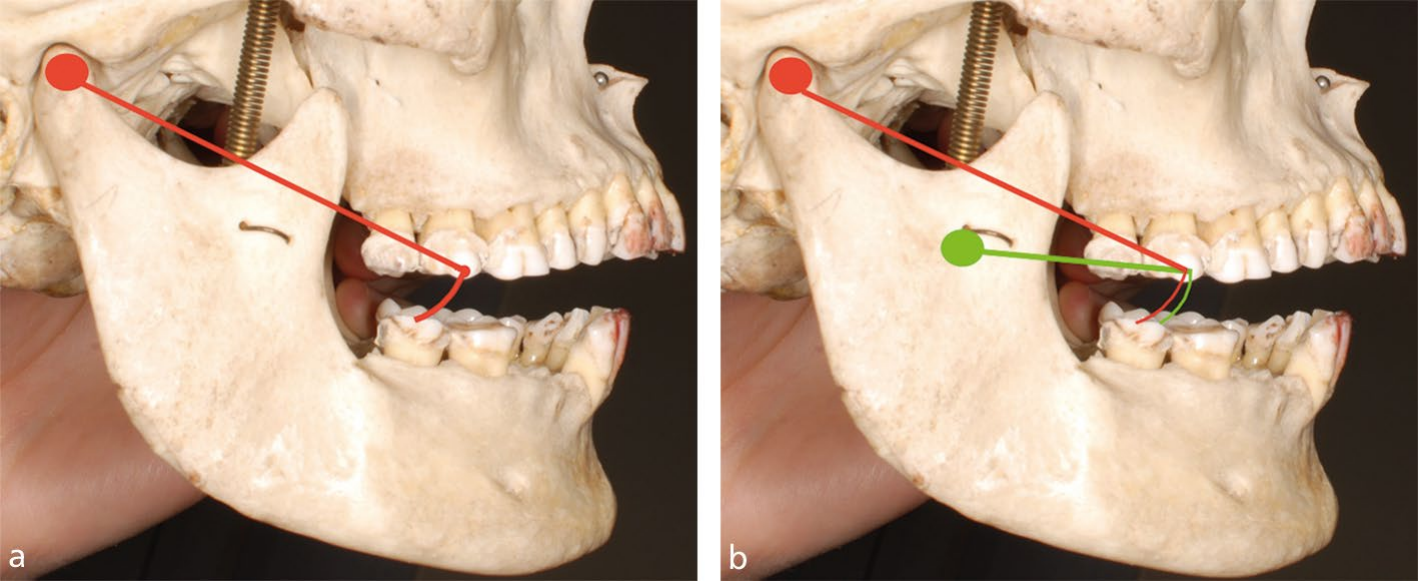
a, b) Diagrams illustrating the different arc of closure the 47 will take depending on where the terminal hinge axis is positioned

Aesthetically, it allows any incisal and occlusal cants, relative to the horizontal reference plane being utilised, to be recorded. Although this can be achieved in other ways, a facebow is quick and predictable (Fig. 9). If this information is not provided, the technician's default mounting position will be to align the occlusal plane to the bench top. The facebow transfer also gives functional benefits. By mounting the maxillary model on the articulator at the same position relative to the terminal hinge axis as is present anatomically, the arc of opening and closing taken by the mandible is replicated on the articulator (Fig. 10). This is important and ensures that the morphology of any new prosthetic teeth take in to account the patient's arc of closure and rotation. This is particularly important if the occlusal vertical dimension is to be opened or closed on the articulator.

### Limitations of facebows

There are many different articulator manufacturers on the market, each with their own specific facebow. It is essential that the correct facebow to the articulators used in your chosen laboratory is selected. Facebows rely on two posterior reference points and one anterior reference point when positioning their upper member. Confusingly, these reference points change depending on the brand of facebow utilised. It is therefore essential that the manufacturer's instructions are followed.

**Fig. 11:**
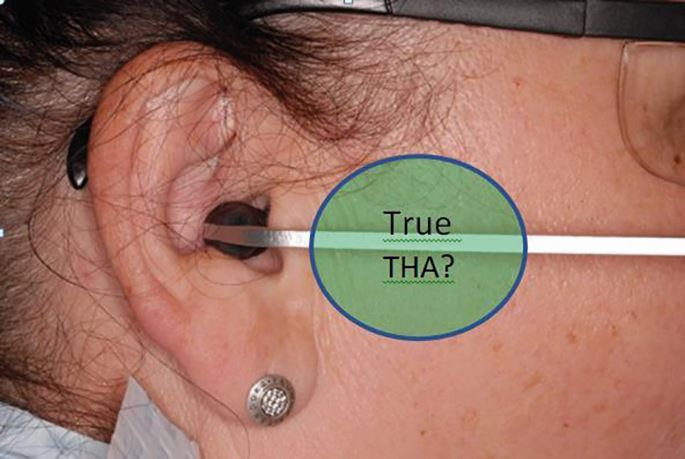
The ear bow is positioned in the external auditory meatus. The external auditory meatus is an arbitrary representation for the true anatomical position of the condyle in the glenoid fossa and the patient's terminal hinge axis. The true position of this patient's terminal hinge axis is unknown

Most facebows utilise the external auditory meatus (EAM) as a convenient, but arbitrary, posterior reference point to represent the terminal hinge axis (THA). The THA is an imaginary line drawn between the patient's condyles around which the mandible rotates in early opening and late closing. Because of this, they are more appropriately named 'earbows' (Fig. 11). The EAM is not the exact position of the THA, but most earbows are accurate to 6 mm.^24^

Manufacturers have gone to great lengths to construct the upper member of their facebow in order to account for the average discrepancy between the anatomical position of the THA and the EAM. But there is no doubt that this 'one size fits all' approach will lead to inaccuracy. The impact of this has been studied by researchers and it has been concluded that even a 5 mm discrepancy between the EAM and the THA will result in only a 0.2 mm discrepancy in mandibular position on closure when a 3 mm thick CCR inter-occlusal record is used.^25^ This discrepancy is unlikely to significantly impact most cases and is a small price to pay for the incredible ease with which the 'earbow' can be used clinically. Greater accuracy can be achieved but involves advanced equipment, such as pantographs and fully adjustable articulators.

When recording the facebow, a non-flexible material, such as silicone bite registration material or beauty wax, should be used to record the tooth positions on the bite fork. It should be possible to stabilise the study cast on the bite fork with three well-distributed points of contact. If this is not possible due to the distribution of the teeth, then a wax record block will be necessary. Ideally, this should be constructed on the model to be mounted to improve accuracy of fit.

## Stage 3: recording a centric relation record

Centric Relation (CR) is a maxillomandibular relationship, independent of tooth position. In CR, the mandible is restricted to a purely rotation movement. Diagnostic casts are mounted on the articulator in CR using a CRR.

### Passive rotation of the mandible

**Fig. 12:**
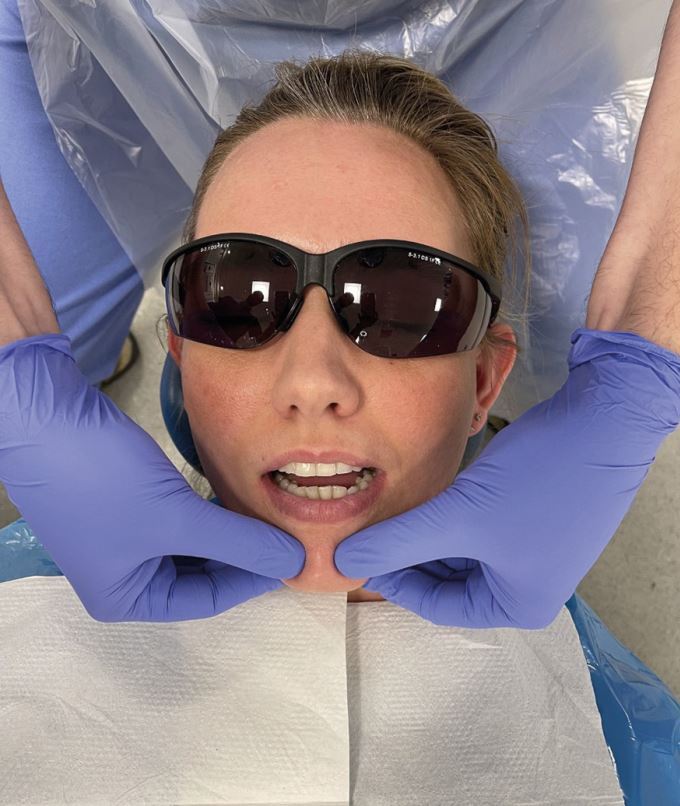
The degree of muscle guarding being assessed in a patient using bi-manual manipulation

The first stage in recording a CRR is to ensure that the clinician is able to reproducibly rotate the patient's mandible with the condyles fully seated. There is debate in the literature about the true anatomic position of the condyle when the mandible is in CR.^26^ But as clinicians, we recognise CR as the familiar feel of 'passive rotation' of the mandible. An accurate CRR can be time-consuming to achieve depending on the degree of muscle guarding the patient has. It is best to test the degree of muscle guarding by gently manipulating the mandible up and down (Fig. 12). There are various techniques to achieve this but the most common is bi-manual manipulation.^27^

**Fig. 13:**
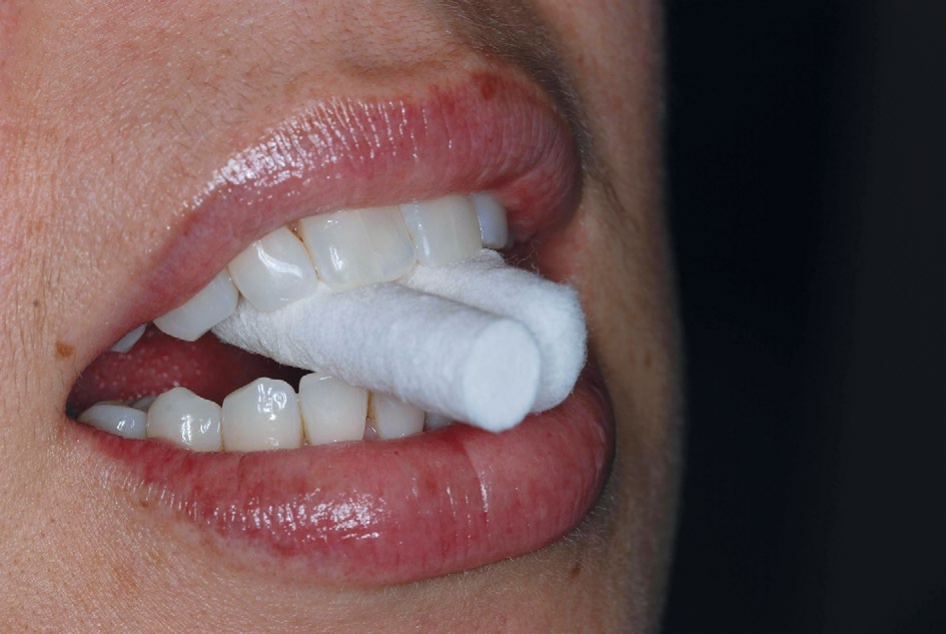
Cotton wool rolls prevent the teeth closing into the inter-cuspal position. Muscle relaxation and predictable rotation of the mandible will often be possible after 2-3 minutes

Passive rotation of the mandible is generally either immediately possible or stunted and difficult. Movement of the patient's head while manipulating the mandible indicates that muscle guarding is present. If muscle guarding is present, then the clinician should seek to achieve muscle relaxation, or deprograming, before proceeding to record the CRR. There is a variety of different techniques that can be used to achieve this and they are best thought of like a ladder of increasing complexity (Box 1). The clinician should only ascend to the next rung as each of the simpler techniques has been exhausted and failed to deprogramme the muscles successfully. The most straightforward technique that can be successful in achieving muscle relaxation is to simply hold the teeth apart for 2-3 minutes. This is easily achieved using cotton wool rolls (Fig. 13). It is essential that during this process, the patient should not be allowed to re-close their teeth into the inter-cuspal position. This will immediately re-introduce a degree of muscle guarding.

**Fig. 14:**
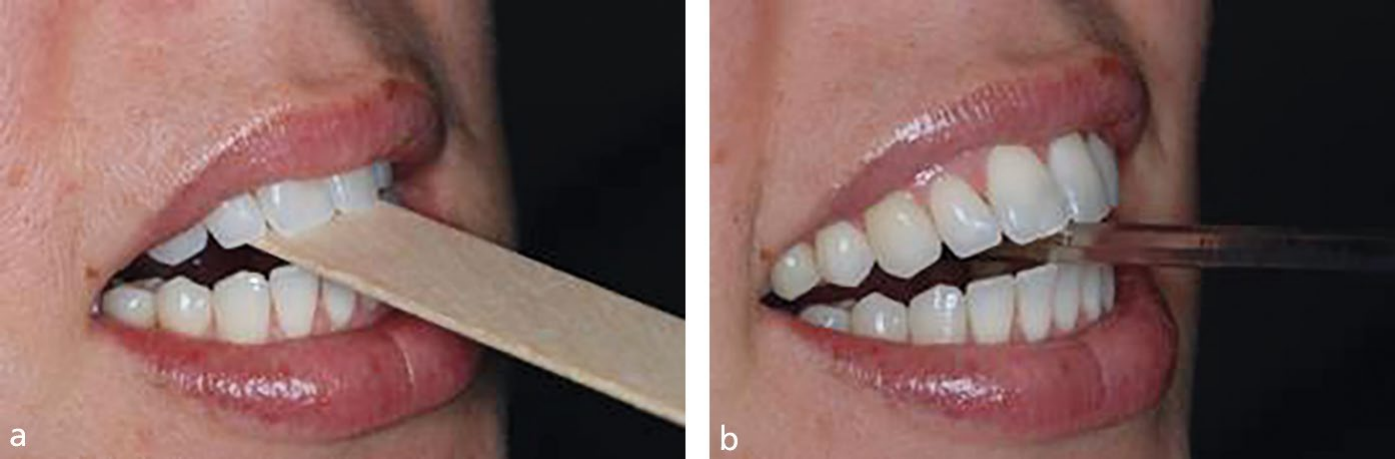
a, b) The patient may need a more dynamic movement of the mandible to achieve muscle relaxation. A mirror handle or wooden spatula is a cheap and quick way to achieve this

In some patients, this simple technique is not enough, and they require more dynamic movement of the mandible to allow relaxation of the muscles. The use of a mirror handle or a small stack of wooden spatulas can be useful, encouraging the patient to slide their mandible forward and back on them (Fig. 14). A leaf gauge can also be useful for this purpose. The use of which is well-explained in published articles.^28^

**Fig. 15:**
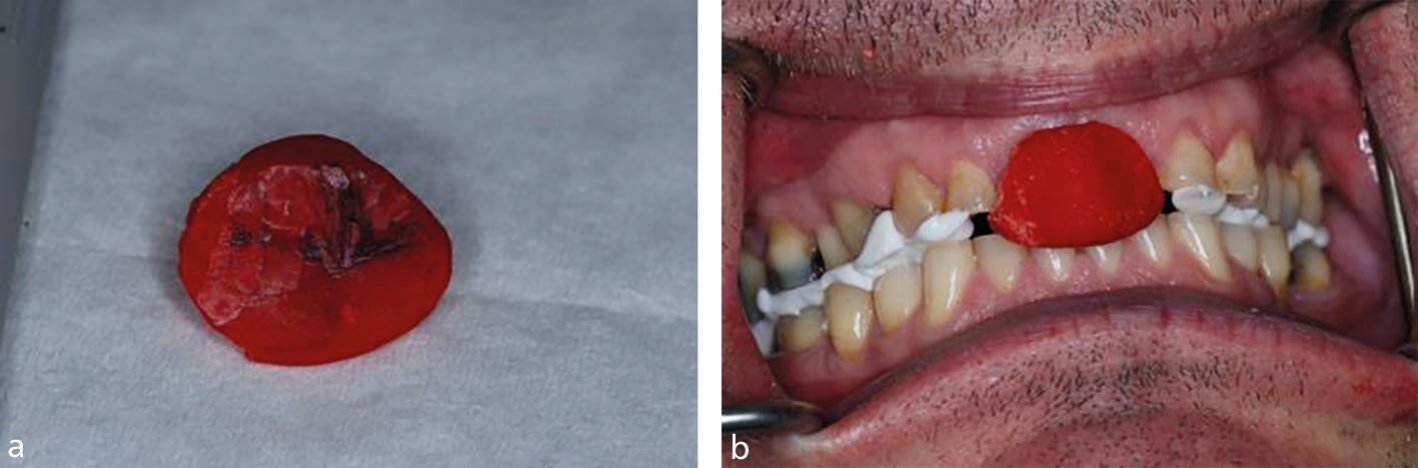
a, b) A Lucia Jig is an excellent tool when muscle relaxation is challenging to achieve. The production of a gothic arch tracing confirms CR has been achieved, demonstrated here at the head of the arrow

If this is unsuccessful a Lucia Jig can be used.^29^^,^^30^^,^^31^ These can be bought prefabricated and relined, or constructed from scratch using pattern resin or cold cure acrylic. The advantage of the Lucia Jig is that it can be left in place for extended periods without direct clinician input. This is hugely advantageous if the patient's muscles need longer to deprogramme. The Lucia Jig will often need adjusting to achieve a single lower incisor contact. The production of an even gothic arch tracing on the Lucia Jig indicates that patient has been deprogrammed (Fig. 15). If the patient is partially dentate, it may not be possible to construct a Lucia Jig and the clinician could consider using a central bearing apparatus as an alternative.

If all of the steps above are exhausted and muscle guarding remains, it may be that longer-term deprograming is needed. This can be achieved with a stabilisation splint. This appliance can be sequentially equilibrated until such time, as consistent occlusal contacts are recorded at multiple review appointments. This is unnecessary in all but the most extreme of cases.

Box 1 A ladder of techniques that can be used to achieve muscle relaxation **Ladder for deprogramming the patient**

Rung 5: Stabilisation splint

Rung 4: Lucia Jig/central bearing apparatus

Rung 3: Leaf gauge

Rung 2: Spatula or mirror handle between the teeth

Rung 1: Cotton wool roll between the teeth

### Recording the centric relation record

**Fig. 16:**
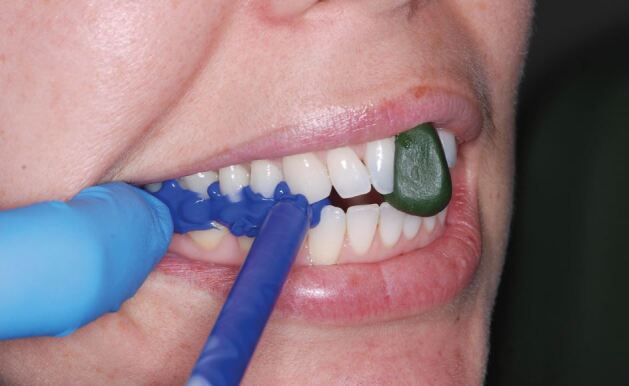
A greenstick stop being utilised to hold the patient's teeth slightly apart in CR as a PVS bite registration material is used to record the CRR

**Fig. 17:**
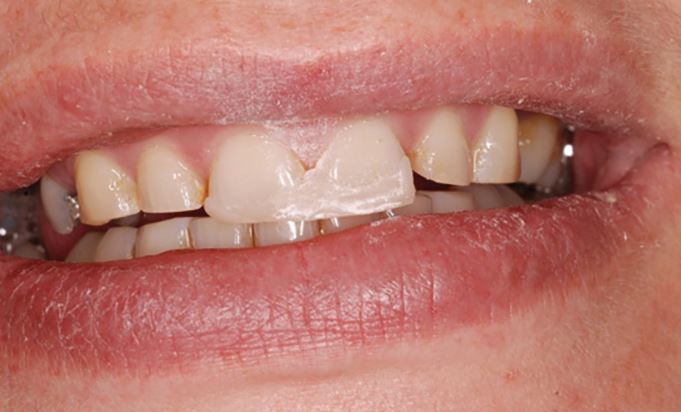
It can be useful to mock the anterior teeth with unbonded composite to represent the proposed tooth wear build-ups. This template can then be used instead of a greenstick stop when recording the CRR

A CRR should be recorded with the maxillary and mandibular teeth apart. It should never be perforated by the occlusal contacts. Traditionally, a trimmed piece of beauty wax has been used to record the CRR. A modern alternative is to use a green stick impression compound stop and poly vinyl silicone (PVS) bite registration material (Fig. 16). It can also be useful to mock the anterior stop in composite to represent where your proposed build-ups will extend to (Fig. 17).

It is important that a rigid PVS material is used, ideally one with a Vickers hardness of D or above. Pink or silver wax are not suitable to record a CRR as they are not rigid at room temperature.^32^

**Fig. 18:**
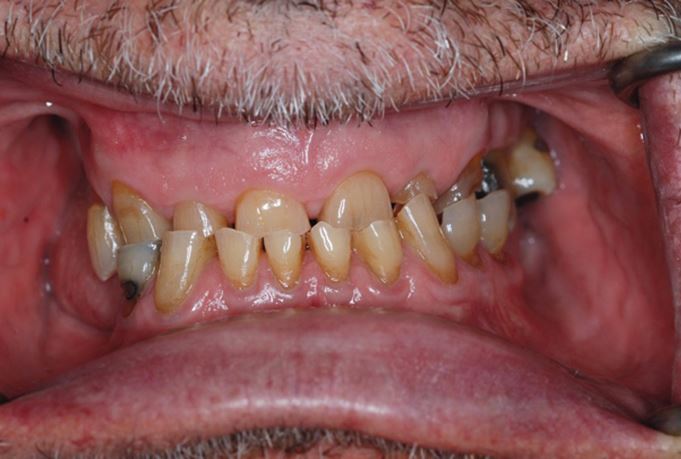
A typical tooth wear case with limited posterior tooth contacts. A wax registration rim will be required in order to achieve an accurate CRR

**Fig. 19:**
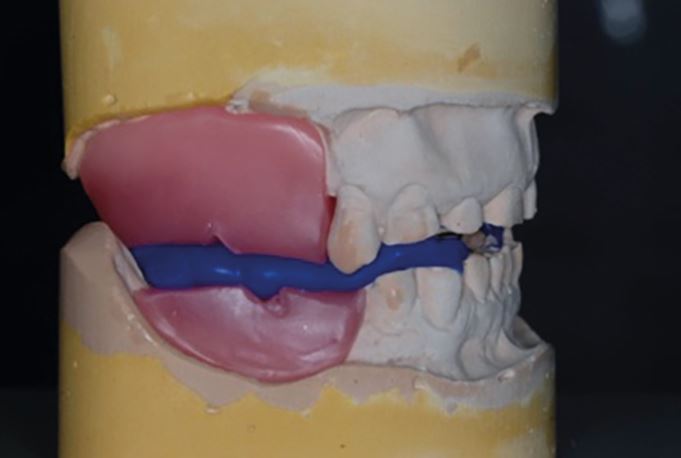
Wax rims have been used as part of the CRR. The wax registration blocks should not come into contact to prevent compression into the mucosa and causing inaccuracies in the mounting process

It is essential that the CRR has a minimum of tripod contacts on the remaining teeth. If the patient has multiple missing posterior teeth, there may be a need to include wax registration rims as part of the CRR (Fig. 18). Wax rims can introduce large errors into the registration process if not managed carefully due to the differential between tooth and mucosa compressibility. Ideally, the wax registration blocks should have been fabricated on the study casts that are to be articulated. It is best to prevent wax registration rims coming into direct contact with each other, with their final vertical position recorded using silicone registration material. This will reduce the risk of them being compressed into the mucosa and rebounding when placed on to a study cast (Fig. 19).

**Fig. 20:**
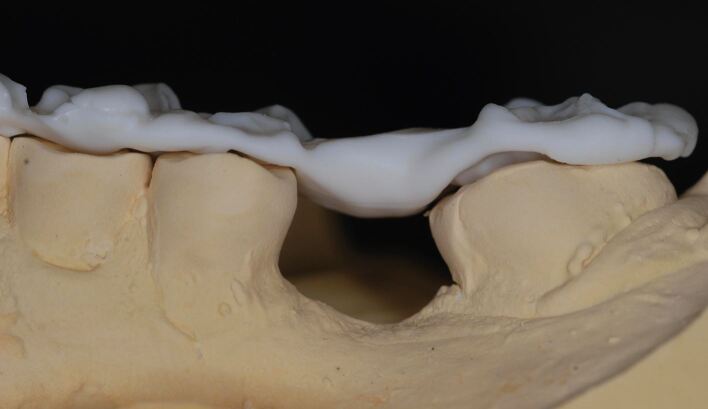
A silicone CRR which is not fully seating due to the mismatch in accuracy between the fissure pattern captured by the silicone and those reproduced by the alginate impression and stone cast

**Fig. 21:**
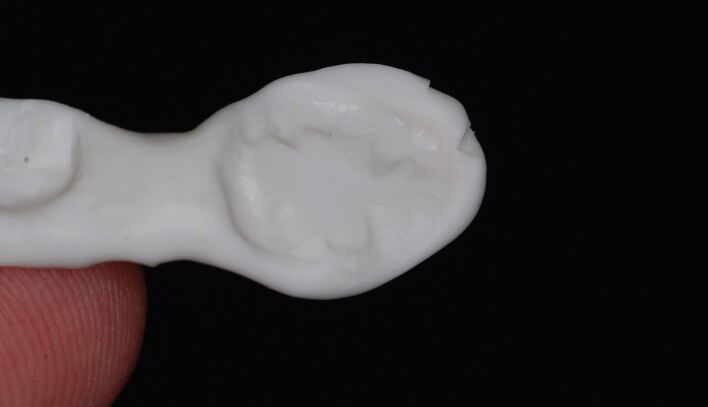
The same CRR which has been cut back to show cusp tips only. By removing the fissures, the silicone CRR will more easily seat down fully

**Fig. 22:**
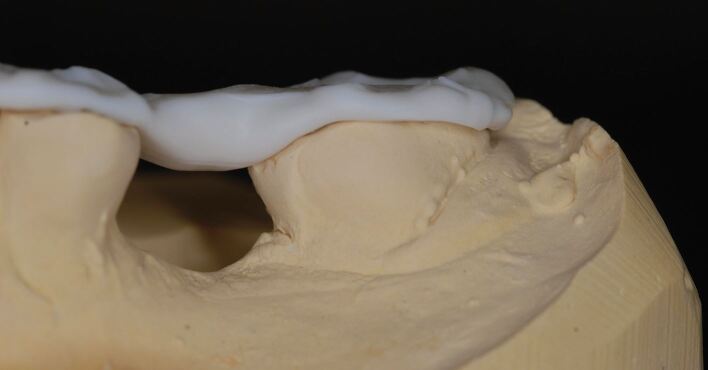
The fully seated CRR after appropriate modification

Silicone bite registration material records more surface detail than alginate. The high degree of accuracy with which the silicone captures the fissure pattern of the teeth prevents the occlusal record from fully seating on the study casts produced from the alginate impressions (Fig. 20). It is therefore essential that the CRR is 'cut back' to show cusp tips only. It is also essential for the same reason that blebs and air blows are removed from the stone model. Without doing so, the registration will not fully seat down (Fig. 21, Fig. 22). It can be advantageous to record the impressions and CRR at separate staged appointments, as this allows the stability of the CRR to be checked in clinic before sending to the laboratory for mounting.

## Stage 4: lab mounting and verification

After the records above have been collected and processed by the laboratory, it is essential that the mounted study casts are verified for accuracy. Accuracy is by no means a guarantee and errors can occur at any point. This can result in mounted study casts that do not replicate the tooth contacts found clinically.

**Fig. 23:**
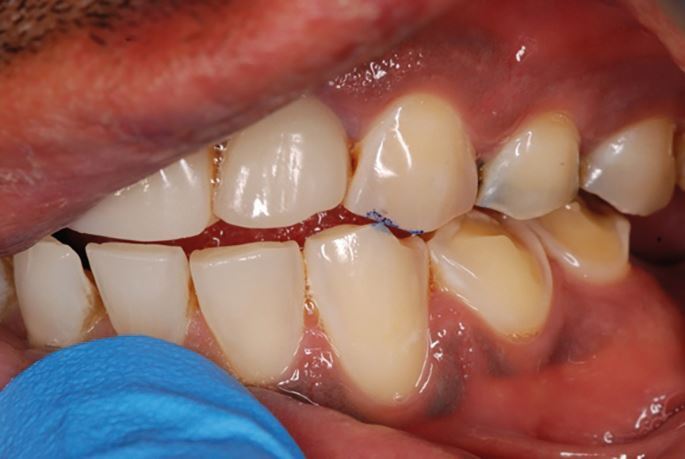
Picture showing the initial tooth contact between the 23 and 33 when the patient's mandible is rotated upwards with condyles fully seated

The occlusion should already have been examined in detail clinically.^33^^,^^34^^,^^35^^,^^36^ Verification can be completed in different ways. The recording of multiple CRRs allows the use of a Centri-Check.^37^ More practically, the initial tooth contact in CR can be marked with articulating paper clinically. Occlusal indicator wax can also be used to record and retain this tooth contact. An occlusal sketch could be used for the same purpose (Fig. 23).

**Fig. 24:**
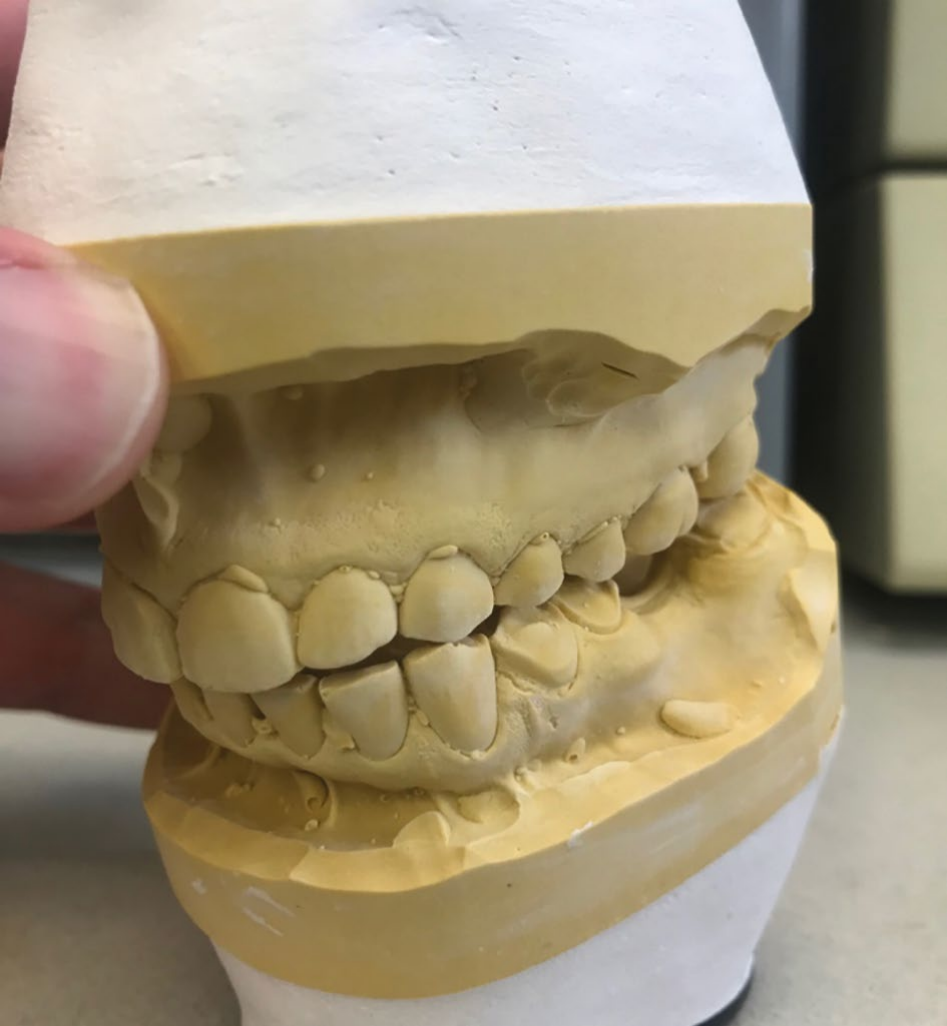
Verification of the occlusal contacts on the articulated study casts is essential. In this case, the initial tooth contact between the 23 and 33 has been reproduced

When the models are returned from the laboratory, the initial tooth contact on the study casts should replicate those found clinically. This can be marked with articulating foil and confirmed with the occlusal indicator wax or occlusal sketch (Fig. 24). If the clinical occlusal contacts are not replicated, then retaking the CRR or remounting the casts may need to be considered.

## Conclusion

The production of accurate articulated study casts is a time-consuming process. Each stage in the process must be executed to the highest standard if we are to be successful.

Alginate provides a cheap and accurate impressions material but must be handled with care to achieve the best results. The use of a facebow is not always necessary, particularly if not increasing the occlusal vertical dimension. But when needed, the ear bow can provide adequate accuracy for most tooth wear cases, even accepting its limitations relative to a pantograph with fully adjustable articulator. The recording of a CRR can be challenging, particularly in patients with extensive muscle guarding. But by using a staged approach to deprogram the patient's muscles, and selection of the correct inter-occlusal material, an accurate record can be achieved . Even with excellent clinical techniques and care, it cannot be assumed that the lab mounting process will progress without fault. The verification of mounting accuracy in comparison to the patient's clinical tooth contacts is essential for success.
